# Enhanced extracellular expression of *Bacillus stearothermophilus* α-amylase in *Bacillus subtilis* through signal peptide optimization, chaperone overexpression and α-amylase mutant selection

**DOI:** 10.1186/s12934-019-1119-8

**Published:** 2019-04-11

**Authors:** Dongbang Yao, Lingqia Su, Na Li, Jing Wu

**Affiliations:** 1 State Key Laboratory of Food Science and Technology, Jiangnan University, 1800 Lihu Avenue, Wuxi, 214122 Jiangsu China; 2 School of Biotechnology and Key Laboratory of Industrial Biotechnology Ministry of Education, Jiangnan University, 1800 Lihu Avenue, Wuxi, 214122 China; 3 International Joint Laboratory on Food Safety, Jiangnan University, 1800 Lihu Avenue, Wuxi, 214122 China

**Keywords:** Alpha-amylase, Signal peptide, Chaperone, Mutant, High-cell-density fermentation

## Abstract

**Background:**

Our laboratory has constructed a *Bacillus stearothermophilus* α-amylase (AmyS) derivative with excellent enzymatic properties. *Bacillus subtilis* is generally regarded as safe and has excellent protein secretory capability, but heterologous extracellular production level of *B. stearothermophilus* α-amylase in *B. subtilis* is very low.

**Results:**

In this study, the extracellular production level of *B. stearothermophilus* α-amylase in *B. subtilis* was enhanced by signal peptide optimization, chaperone overexpression and α-amylase mutant selection. The α-amylase optimal signal peptide (SP_YojL_) was obtained by screening 173 *B. subtilis* signal peptides. Although the extracellular α-amylase activity that was produced by the resulting recombinant strain was 3.5-fold greater than that of the control, significant quantities of inclusion bodies were detected. Overexpressing intracellular molecular chaperones significantly reduced inclusion body formation and further increased α-amylase activity. Error-prone PCR produced an amylase mutant K82E/S405R (AmySA) with enzymatic activity superior to that of AmyS. Expression of the *amySA* gene with the SP_YojL_ while overexpressing molecular chaperones resulted in a 7.1-fold improvement in α-amylase activity. When the final expression strain (WHS11YSA) was cultivated in a 3-L fermenter for 92 h, the α-amylase activity of the culture supernatant was 9201.1 U mL^−1^, which is the highest level that has been reported to date.

**Conclusions:**

This is the first report that describes an improvement of *B. stearothermophilus* α-amylase extracellular production levels in *B. subtilis* using these strategies, and this represents the highest extracellular production level ever reported for α-amylase from *B. stearothermophilus* in *B. subtilis*. This high-level production provides a basis for enhanced industrial production of α-amylase. These extracellular production level improvement approaches are also expected to be valuable in the expression of other enzymes in *B. subtilis*.

**Electronic supplementary material:**

The online version of this article (10.1186/s12934-019-1119-8) contains supplementary material, which is available to authorized users.

## Background

Alpha-amylases (EC 3.2.1.1), which catalyse the hydrolysis of 1,4-glycosidic bonds in starch and related carbohydrates to produce substances with low degrees of polymerization such as glucose, maltodextrin, and oligosaccharides, are widely used in the food, pharmaceutical, detergent, and textile industries [1]. Alpha-amylases can be isolated from a wide range of sources. Those from microbial sources have received increasing attention due to their low production costs, good fermentation stability, and short production time [2]. The α-amylases from *Bacillus* species are the most widely used, with those from *B. subtilis*, *B. licheniformis*, *B. amyloliquefaciens* and *B. stearothermophilu*s having important industrial applications [3].

With the development of DNA recombination technology, the heterologous expression of α-amylases from different sources has been widely studied. Heterologous expression of the α-amylases from *B. licheniformis*, *B. amyloliquefaciens* and *B. stearothermophilus* have been reported in *Escherichia coli*, *B. licheniformis*, *B. subtilis*, and other hosts [4–6]. *B. subtilis,* a Gram-positive bacterium, offers several advantages. Because *B. subtilis* is classified “generally recognized as safe” (GRAS), it can be used when food safety issues are important. In addition, its genetic manipulation is relatively simple and the product is directly secreted into the extracellular medium. For these reasons, *B. subtilis* has been used to produce a variety of heterologous proteins and chemicals. However, heterologous expression of α-amylases from different microbial sources in *B. subtilis* results in very different extracellular production levels. The extracellular α-amylase activities reported for the α-amylases from *B. licheniformis*, *B. stearothermophilus*, *B. amyloliquefaciens* and *Thermococcus* sp. HJ21 expression in *B. subtilis* are 2012 U mL^−1^ [7], 5086 U mL^−1^ [8], 14 μg mL^−1^ [9], and 19.6 U mL^−1^ [10], respectively.

Efforts to improve the level of heterologous protein expression in *B. subtilis* have included optimization of the signal peptide, transport channel level, chaperone protein level, promoter and other factors of the expression and transport systems. The signal peptide, a special amino acid sequence located at the N-terminus of the secreted protein, plays an important role in protein transport. It helps keep the protein precursor in a transportable folding state, targets the protein precursor to the appropriate transport machinery, and assists transmembrane secretion of the protein into the extracellular medium [11]. Brockmeier et al. [12] pointed out that establishing a library of *B. subtilis* signal peptides and using it to screen for the optimal signal peptide for each foreign protein is an important means to improve heterologous extracellular production levels in *B. subtilis*. They used this method to increase extracellular cutinase activity to 4.7 U mL^−1^. Similarly, Zhang et al. [13] screened 114 signal peptides from the Sec-dependent secretion pathway of *B. subtilis* to identify the optimal signal peptide (SP_PhoB_) for heterologous xylanase expression in *B. subtilis*. Using this technique, they increased the extracellular xylanase activity to 193.7 U mL^−1^. In addition, Tsuji et al. [14] screened 173 *B. subtilis* signal peptides to identify the best signal peptide (SP_Pbp_) for heterologous natto phytase expression in *B. subtilis*. The resulting recombinant strain, TSU004, produced twice the natto phytase activity obtained using the control strain TSU003.

Naturally, successful secretion of recombinant proteins from *B. subtilis* requires that intracellular chaperones prevent inappropriate folding or aggregation of the protein precursors within the cell and that the intracellular protein precursors undergo efficient transmembrane transport. There are two types of intracellular chaperones in *B. subtilis* [15]. One type targets protein precursors to membrane transport channels in the cell. Examples of this type include Ffh and FtsY. The other type keeps the protein precursor folded in a state that can be secreted across the membrane. Examples of this type include trigger factor (TF), GroEL, GroES, DnaK, DnaJ, and GrpE. Chen et al. [6] increased the extracellular activity of AmyL by 132%, 133% and 138%, compared with a control, by overexpressing *ftsy*, a partial *dnaK* operon, and *SRP*, respectively, in *B. subtilis*. They also increased extracellular AmyS activity to 124%, 125%, 126% and 149%, compared with a control, by overexpressing *SRP*, *ftsy*, the *groESL* operon, and a partial *dnaK* operon, respectively, in *B. subtilis*.

The α-amylase from *B. stearothermophilus* has received increasing attention due to its high specific activity and acid resistance [16]. Although *B. stearothermophilus* can secrete amylase naturally, the natural extracellular production of amylase produced by *B. stearothermophilus* is relatively low [17]. Therefore, recombinant expression of the amylase is necessary to increase its production [17]. However, *B. stearothermophilus* is not a commonly used expression host strain, its genetic background is not yet clear, and the corresponding molecular manipulation techniques are not yet mature. In addition, the culture temperature of amylase produced by *B. stearothermophilus* is relatively high at approximately 55 °C, which is not conducive to the industrial production of amylase [18]. In contrast, the *B. subtilis* expression system is established, the genetic background is clear, and the corresponding molecular manipulation techniques are relatively mature. Therefore, in this study, the *B. stearothermophilus* amylase was heterologously expressed in *B. subtilis*. In previous studies, heterologous expression of *B. stearothermophilus* α-amylase in *B. subtilis* produced low levels of enzyme activity. Although the overexpression of molecular chaperones, signal peptide optimization and other efforts have increased extracellular production levels, the highest amylase activity in the fermentation supernatant reported to date is only 5086 U mL^−1^ [8]. Because *B. stearothermophilus* amylase has important application value for maltooligosaccharide and high fructose syrup production, and the amylase industrial demand is large, increasing its production is very important to decrease the application cost. Although there are other avenues of research that could prove useful, few related literature reports. For example, few reports describe mutation of the target protein to increase enzyme activity as a way to increase the level of heterologous expression of *B. stearothermophilus* α-amylase in *B. subtilis*. In this study, we combined a signal peptide screening campaign, an intracellular chaperone overexpression strategy, and a random mutagenesis strategy to improve heterologous expression of *B. stearothermophilus* α-amylase in *B. subtilis*. Once an optimal expression strain was constructed, its ability to produce *B. stearothermophilus* α-amylase was verified using a 3-L fermenter.

## Materials and methods

### Bacterial strains and media

All strains used in this study are listed in Table 1. *E. coli* JM109 was used to construct recombinant vectors. *E. coli* BL21 was used to screen for α-amylase mutants with improved α-amylase activity. *B. subtilis* RIK1285 was used to screen signal peptides in α-amylase production. *B. subtilis* WS11 and WHS11 were used as heterologous expression hosts for α-amylase. The α-amylase gene (*amyS*) from *B. stearothermophilus* was used in this study. The LB medium was used as the seed medium, and the shake-flask fermentation was carried out by TB medium. The fermentation medium used in the 3-L fermenter contained (g L^−1^) 15.0 industrial peptone, 15.0 corn syrup, 1.0 (NH_4_)_2_-H-citrate, 2.0 Na_2_SO_3_, 2.68 (NH_4_)_2_SO_4_, and 19.2 K_2_HPO_4_, along with 3.0 mL L^−1^ trace element solution [19]. The feeding medium contained (g L^−1^) 200.0 industrial yeast powder and 300.0 glucose, along with 20.0 mL L^−1^ trace element solution.

**Table 1:** Strains used in this study

Strains	Properties	Reference
Strains		
*E. coli* JM109	*endA1*, *recA1*, *thi*, *gyrA96*, *supE44*, *hsdR17* Δ (*lac*-*proAB*)/F′ [*traD36, lacI*^*q*^*, lacZ*ΔM15, *proAB*^+^]	Takara
*E. coli* BL21	F^−^, ompT, hsd SB (rB^−^mB^−^), gal, dcm	Takara
*B. subtilis* RIK1285	Marburg 168 derivative: *trpC2*, *lys1*, *aprE Δ3*, *nprR2*, *nprE18*	Takara
*B. subtilis* RIK1285AS	*B. subtilis* RIK1285 containing plasmid pBE-SP_AprE_-*amyS*	This study
*B. subtilis* WS11	WS4 derivative, Δ*wprA*, Δ*nprB*, Δ*vpr*, Δ*epr*, Δ*bpr*, Δ*mpr*, Δ*aprE*	[23]
*B. subtilis* WHS11	WS11 derivative, Δ*hrcA*	Our laboratory
WS11QS	*B. subtilis* WS11 containing plasmid pHYQamyS	This study
WS11QSP	*B. subtilis* WS11 containing plasmid pHYQamySP	This study
WS11RS	*B. subtilis* WS11 containing plasmid pHYRamyS	This study
WS11AS	*B. subtilis* WS11 containing plasmid pHYAamyS	This study
WS11YS	*B. subtilis* WS11 containing plasmid pHYYamyS	This study
WS11YSP	*B. subtilis* WS11 containing plasmid pHYYamySP	This study
WHS11YS	*B. subtilis* WHS11 containing plasmid pHYYamyS	This study
WHS11YSA	*B. subtilis* WHS11 containing plasmid pHYYamySA	This study

### Plasmids construction and transformation

All plasmids and primers used in this study are listed in Table 2 and Additional file 1: Table S1, respectively. The α-amylase gene *amyS* was amplified from pET-20b-*amy* [4] using primers pBE1 and pBE2 and then cloned into the vector pBE-S DNA using restriction enzymes *Sac*I and *Hin*dIII, creating the plasmid pBE-SP_aprE_-*amyS*. The pBE-*amyS* fragment (backbone of pBE-SP_mix_-*amyS*) was obtained from pBE-SP_aprE_-*amyS* using primers pBE3 and pBE4. The isolated pBE-*amyS* fragment was linked with 173 different *B. subtilis*-derived secretory signal peptides (purchase from Takara) using Takara’s In-Fusion HD Cloning kit. The pHYQ fragment was amplified from plasmid pHYCGTd4 [19] using primers pHY1 and pHY2. The α-amylase gene *amyS* was amplified from pET-20b-*amy* using primers pHY3 and pHY4 and then joined with the pHYQ fragment using Takara’s In-Fusion HD Cloning kit to create pHYQamyS. The pHY fragment was amplified from plasmid pHYQamyS using primers pHY5 and pHY6. The SP_RpmG_-*amyS*, SP_ApsB_-*amyS*, and SP_YojL_-*amyS* fragments were amplified from plasmids pBE-SP_RpmG_-*amyS*, pBE-SP_ApsB_-*amyS* and pBE-SP_YojL_-*amyS*, respectively, using forward primers pHY7, pHY8 and pHY9, respectively, and reverse primer pHY10. These fragments were joined with the pHY fragment using Takara’s In-Fusion HD Cloning kits to create pHYRamyS, pHYAamyS, and pHYYamyS, respectively. The pHYQamyS and pHYYamyS fragments (backbones of pHYQamySP and pHYYamySP, respectively) were amplified from plasmids pHYQamyS and pHYYamyS, respectively, using primers pHY11 and pHY12. The P_apr_-*prsA* fragment was amplified from plasmid pHYPULd4P using primers pHY13 and pHY14 and then joined with the pHYQamyS and pHYYamyS fragments using Takara’s In-Fusion HD Cloning kit to create pHYQamySP and pHYYamySP, respectively. Using the method of Anagnostopoulos and Spizizen [20], these protein expression vectors were transformed into *B. subtilis*. A library of mutant α-amylase genes (*amyS*′) was amplified from pET-20b-*amy* with primers pF1 and pR1 using error-prone PCR [21] and then cloned into the vector pET24a(+) using restriction enzymes *Nco*I and *Hin*dIII, creating pET24a-*amyS*′. The carrier pET24a(+) had been modified in our laboratory with a pelB signal peptide. The recombinant vector pHYYamySA was obtained by replacing *amyS* in the recombinant vector pHYYamyS with the mutant K82E/S405R (*amySA*).

**Table 2:** Plasmids used in this study

Plasmids	Properties	References
pBE-S DNA	Amp^r^ (*E. coli*), Kan^r^ (*B. subtilis*), P_aprE_, SP_aprE_	Takara
pBE-SP_mix_-*amyS*	Amp^r^ (*E. coli*), Kan^r^ (*B. subtilis*), P_aprE_, SP_mix_, α-amylase gene *amyS*	This study
pBE-SP_AprE_-*amyS*	Amp^r^ (*E. coli*), Kan^r^ (*B. subtilis*), P_aprE_, SP_aprE_, α-amylase gene *amyS*	This study
pBE-SP_RpmG_-*amyS*,	Amp^r^ (*E. coli*), Kan^r^ (*B. subtilis*), P_aprE_, SP_RpmG_, α-amylase gene *amyS*	This study
pBE-SP_ApsB_-*amyS*	Amp^r^ (*E. coli*), Kan^r^ (*B. subtilis*), P_aprE_, SP_ApsB_, α-amylase gene *amyS*	This study
pBE-SP_YojL_-*amyS*	Amp^r^ (*E. coli*), Kan^r^ (*B. subtilis*), P_aprE_, SP_YojL_, α-amylase gene *amyS*	This study
pET-20b-*amy*	Amp^r^ (*E. coli*), α-amylase gene	[4]
pET24a-*amyS*′	Kan^r^ (*E. coli*), random mutant of α-amylase gene	This study
pET24a-*amySA*	Kan^r^ (*E. coli*), α-amylase gene mutant amyS(K82E/S405R)	This study
pHYCGTd4	Amp^r^ (*E. coli*), Tet^r^ (*E. coli* and *B. subtilis*), dual promoter P_HpaII_-P_amyQ′_, SP_AmyQ′_, β-CGTase gene	[19]
pHYQamyS	pHYCGTd4 derivative, SP_AmyQ′_, α-amylase gene *amyS*	This study
pHYRamyS	pHYCGTd4 derivative, SP_RpmG_, α-amylase gene *amyS*	This study
pHYAamyS	pHYCGTd4 derivative, SP_ApsB_, α-amylase gene *amyS*	This study
pHYYamyS	pHYCGTd4 derivative, SP_YojL_, α-amylase gene *amyS*	This study
pHYPULd4P	Amp^r^ (*E. coli*), Tet^r^ (*E. coli* and *B. subtilis*), dual promoter P_HpaII_-P_amyQ′_, P_apr_, β-CGTase gene, PrsA gene *prsA*	Our laboratory
pHYQamySP	PHYQamyS derivative, PrsA gene *prsA*	This study
pHYYamySP	PHYYamyS derivative, PrsA gene *prsA*	This study
pHYYamySA	PHYYamyS derivative, α-amylase gene *amySA*	This study

### Cultivation conditions

#### 96-well plate cultivation

Seed solutions were prepared by adding single colonies to the wells of 96-well plates containing 600 μL of LB medium supplemented with 30 mg L^−1^ kanamycin; the inoculated plates were cultured at 37 °C and 900 rpm for 8 h. Aliquots of these seed cultures (50 μL) were then transferred to a new 96-well plate containing 600 μL TB medium supplemented with 30 mg L^−1^ kanamycin. These plates were incubated at 37 °C and 900 rpm for 2 h, then at 33 °C and 900 rpm for 48 h.

#### Shake flask cultivation

Seed cultures were started by inoculating 10 mL of LB medium supplemented with 30 mg L^−1^ of the appropriate antibiotic in a 50 mL shake flask with a 20 μL sample of frozen glycerol stock (stored at − 80 °C). These cultures were incubated at 37 °C and 200 rpm for 8 h. Aliquots of the seed culture (5% [v/v]) were then transferred to a 250 mL shake flask containing 50 mL TB medium supplemented with 30 mg L^−1^ of the appropriate antibiotic. These cultures were incubated at 37 °C and 200 rpm for 2 h, and then at 33 °C and 200 rpm for 48 h. The culture supernatant, which was used as the crude enzyme solution, was obtained by centrifugation at 12,000*g* for 15 min at 4 °C.

#### 3-L Bioreactor cultivation

Seed cultures was started by inoculating 50 mL of LB medium in a 250 mL shake flask with a 100 μL sample of frozen glycerol stock (stored at − 80 °C). The resulting cultures were incubated at 37 °C and 200 rpm for 10 h. In a 3-L fermenter (Labfors, Infors-HT Co., Ltd), the fermentation medium was inoculated with seed cultures in a ratio of 10% (v/v), and 20 mg L^−1^ tetracycline was added to the fermentation medium every 24 h. After inoculation, the fermentation was carried out at 37 °C, the pH was maintained at 7.0 using 20% (v/v) H_3_PO_4_ and NH_4_OH, and the dissolved oxygen (DO) was maintained at approximately 30% by cascading the agitation speed (200 to 800 rpm) and injecting air mixed with pure oxygen. After approximately 8 h of culture, the DO suddenly increased and the agitation speed suddenly dropped, indicating that the initial glucose was completely consumed. At this point, fed-batch cultivation was initiated. The concentration of glucose in the medium was measured by SBA-40C biosensor (Biology Institute of Shandong Academy of Sciences, Jinan, China) and was maintained below 0.5 g L^−1^ by adjusting the feeding rate. At defined time intervals, the medium was sampled. The culture supernatant, which was used as the crude enzyme solution, was obtained by centrifugation at 12,000*g* for 15 min at 4 °C.

### Determination of bacterial biomass

To determine the dry cell weight (DCW), a 10 mL sample of the culture solution was centrifuged at 12,000*g* and 4 °C for 15 min. After removing the supernatant, the cell pellet was washed by resuspending it in 0.9% (w/v) NaCl solution and centrifuging at 12,000*g*, 4 °C for 15 min. After repeating the wash with 0.9% (w/v) NaCl solution, the supernatant was removed and the cell pellet was dried 105 °C to constant weight.

### Construction of signal peptides library and α-amylase mutants library

Takara’s *B. subtilis* “Secretory Protein Expression System” and 173 *B. subtilis* signal peptides were used to identify the optimal signal peptide for α-amylase. *B. subtilis* RIK1285 was transformed with recombinant vector pBE-SP_aprE_-*amyS* to construct the control strain *B. subtilis* RIK1285AS. The signal peptide screening library was constructed by replacing signal peptide SP_aprE_ in the recombinant vector pBE-SP_aprE_-*amyS* with 173 different *B. subtilis*-derived secretory signal peptides, and then using this library of vectors to transform *B.* subtilis RIK1285. A library of strains expressing random α-amylase mutants was constructed by transforming *E. coli* BL21 with the vector pET24a-*amyS*′.

### Enzyme activity assays and purification of α-amylase

Qualitative screening of the library of random α-amylase mutants was performed by cultivating individual colonies in 96-well plates and then using the culture supernatants to inoculate 1% agar plates containing 2% soluble starch. Assay plates were incubated at 60 °C for 90 min, and then the α-amylase activity was evaluated by measuring the diameters of the clearance zones after staining with iodine. First, a clear circle larger than that produced by the control strain was selected by visual observation; then, the diameters of the two were measured with a ruler, and the ratio of the diameter of the selected clear circle to the diameter of the control clear circle was calculated. When the ratio was greater than 1.5, the larger the value, the higher the fermentation supernatant amylase activity of the strain corresponding to the clear circle. When qualitatively measuring amylase activity, each sample was tested three times, and the data were analysed according to the methods in “Statistical analysis” section of “Materials and methods”. A quantitative α-amylase activity assay was performed by measuring the amount of reducing sugar released from soluble starch. The amount of reducing sugar was determined using the 3,5-dinitrosalicylic acid (DNS) method [22]. Briefly, a 2.0 mL assay mixture containing 0.1 mL of crude enzyme extract and 1.0 mL 1% soluble starch in 20 mM sodium phosphate buffer (0.9 mL with pH 6.0) was incubated at 70 °C for 5 min, at which point 3 mL of DNS solution was added to terminate the reaction. This reaction mixture was boiled for 7 min; then, 10 mL of deionized water was added, and the absorbance of the mixture at 540 nm was measured. One unit of α-amylase activity was defined as the amount of enzyme that released 1 μmol of reducing sugar per min from soluble starch under the assay conditions described above. Purification of the wild-type (AmyS) and mutant (AmySA) enzymes was performed according to the method described by Li et al. [4]. The optimal temperatures of AmyS and AmySA were determined by assaying protein samples at 60–90 °C in 20 mM phosphate buffer (pH 6.0). The optimal pH of AmyS and AmySA were determined by measuring the enzyme activity at 70 °C in 20 mM citrate buffers (pH 4.5–6.0) and 20 mM phosphate buffers (pH 5.5–7.5), respectively. The enzyme activities at different temperatures and pH values were expressed as a percentage of the highest activity.

The temperature stability of AmyS and AmySA were determined at 90 °C in 20 mM phosphate buffer, pH 6.0, containing 1 mM Ca^2+^. Timed aliquots were removed from incubations; after centrifugation of sample at 12,000*g* and 4 °C for 15 min, the α-amylase enzyme activity present in the supernatant was measured. The sample enzyme activity values of other sampled times were expressed as a percentage of the 0 h sample activity.

### SDS-PAGE gel electrophoresis

The crude enzyme solution containing the target protein was mixed with 5× protein loading buffer, boiled in boiling water for 10 min, and then analysed using sodium dodecyl sulfate-polyacrylamide gel electrophoresis (SDS-PAGE) with a 10% separation gel. Prior to cell disruption, the cell paste was diluted to a density (OD_600_) of 5 and treated with lysozyme for 30 min. The cells were disrupted with an Ultrasonic Homogenizer (Ningbo Xinzhi Biotechnology Co., Ltd. Ningbo, China) for 10 min at 20% power using the cycle: work 2 s, stop 3 s. After centrifugation at 12,000*g* for 15 min at 4 °C, the supernatant was thoroughly separated from the pellet fragments. A 20 μL sample of the supernatant and 5 μL of 5× protein loading buffer were boiled for 10 min; then, an 8 μL portion of this mixture was used for electrophoresis as the cell disruption supernatant. A 20 μL aliquot of 5× protein loading buffer was added to the bacterial debris, and the resulting mixture was boiled for 10 min. After centrifugation at 12,000*g* for 3 min at 4 °C, a 3 μL portion was used for electrophoresis as the cell disruption precipitate. Coomassie Brilliant Blue R-250 was used to stain protein bands.

### Statistical analysis

All experiments were conducted three times, and the data are presented as the averages ± standard deviation. Statistical analyses were conducted using a t test, and the variance at P < 0.05 was considered statistically significant.

## Results and discussion

### Heterologous expression of *B. stearothermophilus* α-amylase in *B. subtilis* WS11

**Fig. 1:**
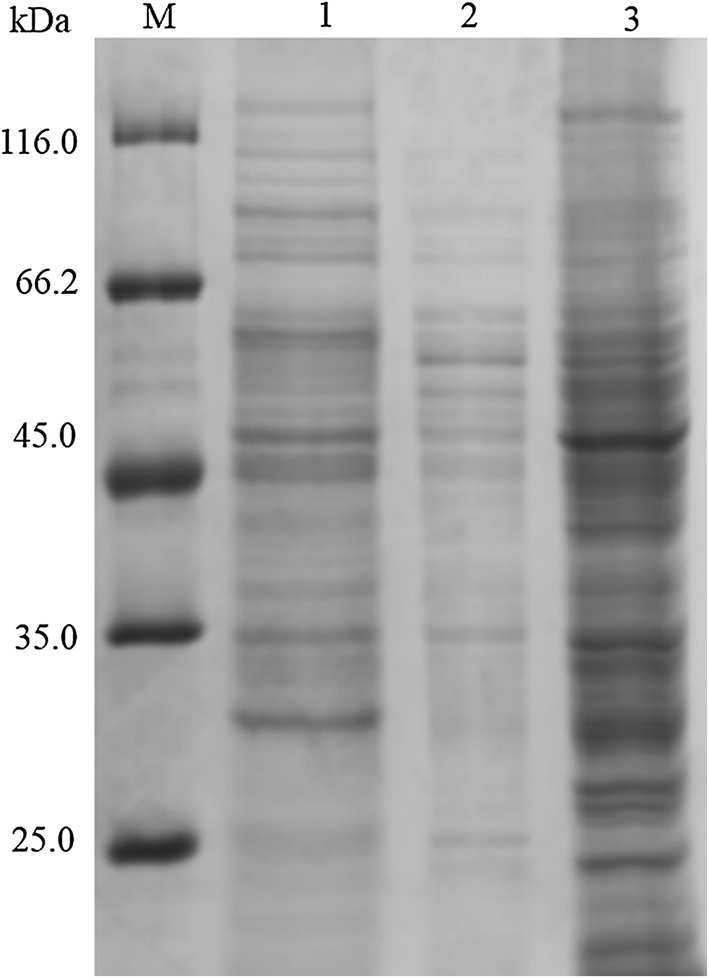
Expression and secretion of α-amylase by *B. subtilis* WS11QS. SDS-PAGE analysis of the culture supernatants (lanes 1), cell disruption supernatants (lanes 2), and cell disruption precipitates (lanes 3) obtained using *B. subtilis* strains WS11QS. Lane M contains the medium molecular weight protein standard

To produce *B. stearothermophilus* α-amylase (AmyS) in *B. subtilis*, the expression strain WS11QS was constructed by transforming *B. subtilis* WS11 with the recombinant vector pHYQamyS, which contained the α-amylase gene *amyS*. WS11QS was cultured in a 250 mL triangular shake flask containing 50 mL of TB medium for 2 h at 37 °C, and then shifted to 33 °C for 48 h to produce α-amylase. The α-amylase activity present in the culture supernatant was 210.4 U mL^−1^. To investigate the distribution of AmyS within WS11QS, cultured cells were treated with lysozyme for 30 min, and then disrupted using a sonicator. SDS-PAGE analysis showed that the culture supernatant, the disruption supernatant, and the disruption precipitate obtained from WS11QS had no obvious bands near 55 kDa, which corresponds to the molecular weight of the α-amylase protein (Fig. 1). This indicates that the expression level of α-amylase in the recombinant WS11QS was very low.

### Isolation and identification of optimal signal peptides for α-amylase

**Fig. 2:**
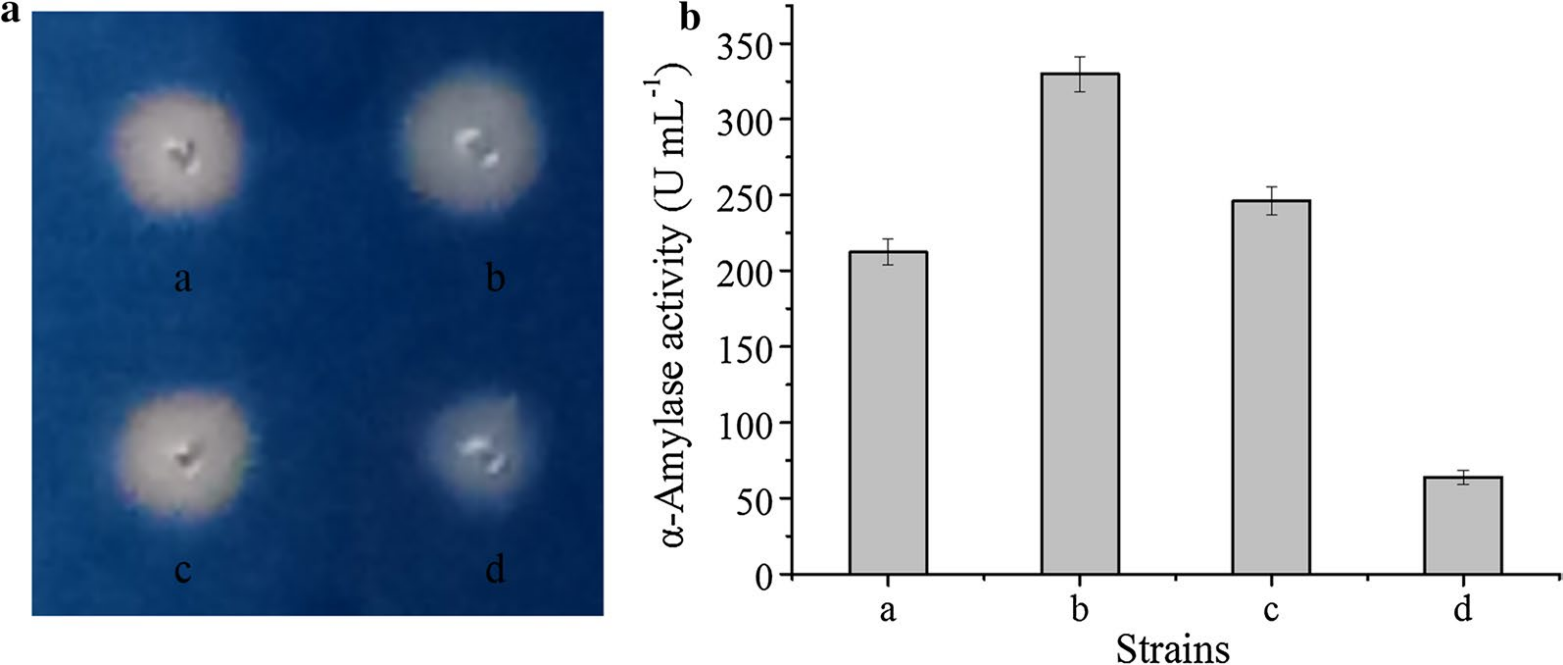
Screening of α-amylases containing different signal peptides. **a** Transparent circles formed on a soluble starch plate by *B. subtilis* RIK1285 strains containing α-amylase variants with different signal peptides. **b** Alpha-amylase activities of the extracellular supernatants obtained using *B. subtilis* RIK1285 strains cultured in a triangular shake flask. Strains a, b, and c represent three *B. subtilis* RIK1285 variants that produce α-amylase with different signal peptides; d is a control strain. Error bars indicate the standard deviation from the mean of the three experimental data replicates

To determine which *B. subtilis* signal peptide would be the best to use with AmyS, N-terminal fusions of AmyS with 173 different *B. subtilis* signal peptides were produced using a commercial screening kit. Individual strains were cultured in 96-well plates, and the activity of AmyS present in the culture supernatants were evaluated by comparing the diameters of the clear circles formed by the culture supernatants on soluble starch plates. After screening 1200 strains, the three strains with the largest transparent circle diameters were selected and fermented in a triangular shake flask to verify their ability to produce α-amylase (Fig. 2a). After 48 h of shake-flask culture, the α-amylase activities of strains a, b, c and control strain d were 212.4, 329.9, 246.1, and 63.9 U mL^−1^, respectively (Fig. 2b). The signal peptides present in strains a–c, identified by sequencing of the expression plasmids, were SP_YojL_, SP_RpmG_, and SP_AspB_, respectively (Table 3).

**Table 3:** Characteristics of selected signal peptides

Signal peptides	Protein sequence	Type	SPase
SP_RpmG_	MRKKITLACKTCGNRNYTTMKSSASA	Sec	SPase I
SP_AspB_	MKLAKRVSALTPSTTLAITAKA	Sec	SPase I
SP_YojL_	MKKKIVAGLAVSAVVGSSMAAAPAEA	Sec	SPase I

**Fig. 3:**
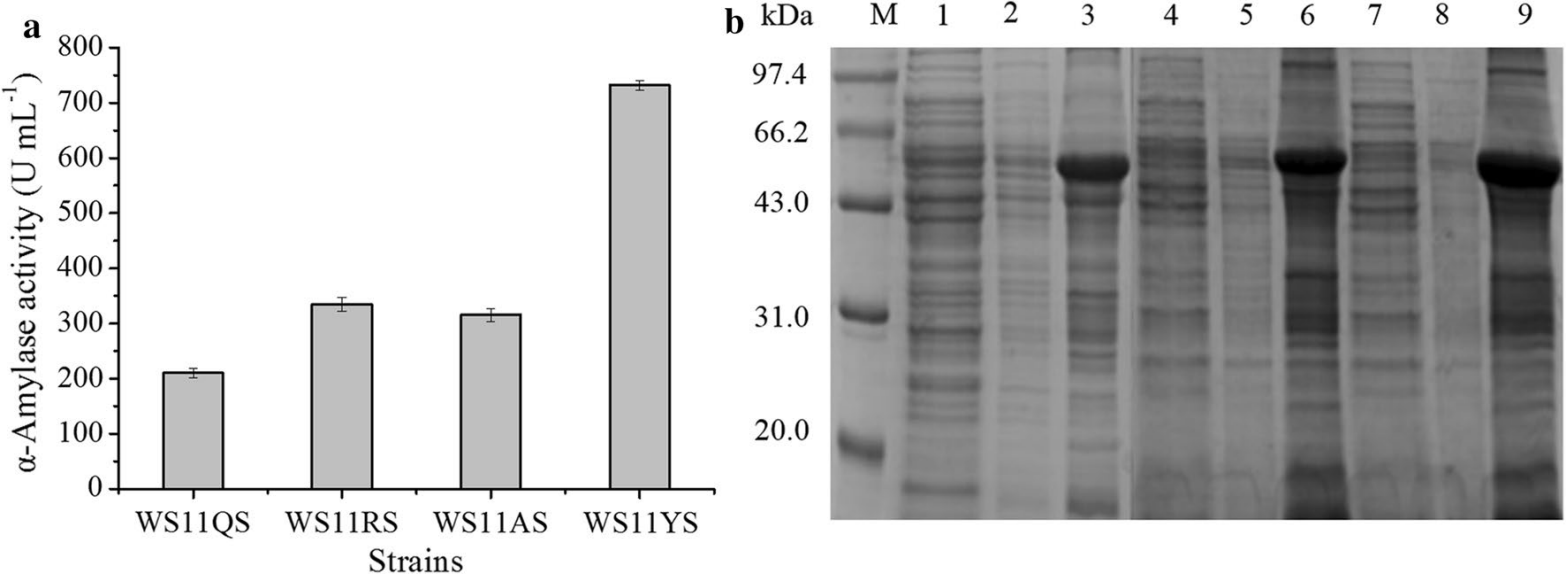
Expression and secretion of α-amylase by *B. subtilis* WS11 variants. **a** Alpha-amylase activity in the shake-flask culture supernatants obtained using *B. subtilis* WS11 variants. **b** SDS-PAGE analysis of the culture supernatants (lanes 1, 4, and 7), cell disruption supernatants (lanes 2, 5, and 8), and cell disruption precipitates (lanes 3, 6, and 9) obtained using *B. subtilis* strains WS11RS, WS11AS, and WS11YS, respectively. Lane M contains the medium molecular weight protein standard. Error bars indicate the standard deviation from the mean of the three experimental data replicates

To further characterize the levels of AmyS produced using each of these signal peptides, we introduced them into an expression system previously constructed in our laboratory [23]. This system uses the expression plasmid pHYCGTd4, which contains the dual promoters P_HpaII_ and P_AmyQ’_, and the expression strain *B. subtilis* WS11, which has had the genes encoding eight proteases (*wprA*, *nprB*, *vpr*, *epr*, *bpr*, *aprE*, *nprE* and *mpr*) deleted from its genome to minimize the degradation of exogenous recombinant proteins. The results of this effort, recombinant strain WS11RS, WS11AS, and WS11YS, express AmyS fused to the signal peptides SP_RpmG_, SP_AspB_ and SP_YojL_, respectively, was constructed. After 48 h of cultivation in a triangular shake flask, the α-amylase activities of the extracellular supernatants of WS11RS, WS11AS, and WS11YS were 334.3, 315.1, and 732.0 U mL^−1^, respectively (Fig. 3a). In contrast to the results obtained with WS11QS (Fig. 1), SDS-PAGE analysis of the cell disruption precipitates of the WS11RS, WS11AS, and WS11YS showed obvious inclusion bodies (Fig. 3b). Because the culture supernatant of the recombinant strain WS11YS had by far the highest enzyme activity, this was the only strain studied in subsequent experiments.

To determine the location of the inclusion bodies formed in WS11YS, their N-terminal amino acid sequence was determined. The amino acid sequence obtained, Met–Lys–Lys–Lys–Ile (Additional file 1: Figure S1), was consistent with the first five amino acids of the signal peptide SP_YojL_ (Table 3). This indicates that the inclusion bodies are formed within the cell by aggregation of the α-amylase protein precursor.

The efficiency of protein precursor transmembrane transport is related to the amino acid sequences of the signal peptide and the mature protein, as well as their interaction with related intracellular elements [24–27]. Despite substantial effort, the specific relationship between signal peptide and target protein remains poorly understood [28]. For a specific protein, a signal peptide with higher hydrophobicity [29], stronger α-helix preference of the amino acid at the end of the H domain [15], and higher N-terminal amino acid charge, generally gives higher translocation efficiency. Because signal peptide SP_YojL_ displays poorer hydrophobicity, α-helix preference of the amino acid at the end of the H domain, and positive charge of the N-terminal amino acid than signal peptide SP_AmyQ′_ (Table 4), it is reasonable to predict that for the α-amylase protein, SP_YojL_ would provide lower transmembrane efficiency, which refers to the rate of amylase precursor transfer to the outer side of the plasma membrane through the plasma membrane channel under the guidance of a signal peptide, than signal peptide SP_AmyQ′_. Thus, the greater extracellular amylase activity caused by SP_YojL_, compared with that obtained using SP_AmyQ′_, is likely associated with a decrease in the intracellular transmembrane transport rate of the corresponding amylase protein precursors.

**Table 4:** Characteristics of signal peptides SP_AmyQ′_ and SP_YojL_

Signal peptides	Hydrophobicity^a^	α-Spiral preference^b^	N domain charge^c^
SP_AmyQ′_	41.5	1.41	53.84
SP_YojL_	33	0.34	34.96

^a^Hydrophobicity: The value of the sum of the hydrophobicity of the H domain was calculated using the ProtScale tool (https://web.expasy.org/protscale/pscale/Hphob.Doolittle.html) [42]. The higher the value, the stronger the hydrophobicity

^b^α-Spiral preference: The conformational preference of amino acids was determined using the tables in *Proteins Structure and Function* [43]. The higher the value, the stronger the preference of the α-spiral

^c^N domain charge: The sum of the charges on the N-terminal amino acid of the signal peptide was calculated from the sum of the isoelectric points of the N-terminal amino acids

Caspers et al. [30] reported a similar phenomenon. They performed saturation mutagenesis of the amino acids in positions 2–7 of the signal peptide region of *B. subtilis* α-amylase and used this sequence to direct the extracellular expression of cutinase from *F. solani pisi* in *B. subtilis.* They found that some of the signal peptide mutants increased the production of *F. solani pisi* cutinase in the growth medium. Pulse-chase experiments with the corresponding protein precursors revealed that some of the cutinase protein precursors containing these mutant signal peptides crossed the membrane at a lower rate.

Sarvas et al. [31] suggested that highly efficient targeting and translocation of protein precursors to the cell membrane transport system may overload PrsA, a molecular chaperone attached to the cytoplasmic membrane of *B. subtilis* [32]. Many studies have shown that PrsA promotes α-amylase folding in *B. subtilis*, thereby increasing α-amylase extracellular production levels [33]. Convinced that the number of PrsA molecules on the cell membrane should be sufficient [9], Yan and Wu [34] suggested that some of the PrsA molecules attached to the cell membrane are far from the Sec-dependent secretion system; otherwise, there would be no need to have so many PrsA molecules. In other words, we can speculate that the effective amount of PrsA attached to the cell membrane, the amount that interacts with the Sec-dependent secretion pathway, is insufficient. Therefore, when the intracellular protein precursor is secreted faster, the effective amount of PrsA is insufficient, causing the incorrectly folded protein to accumulate on the outer side of the cell plasma membrane. This accumulation causes the CrssRS reaction, which induces the production quality control proteases, HtrA and HtrB [35]. These proteases then degrade the α-amylase secreted by the Sec-dependent secretion pathway. However, when the secretion of the intracellular protein precursor is slower, the effective amount of PrsA is relatively sufficient. Under these conditions, the decreased accumulation of incorrectly folded protein on the outer side of the plasma membrane weakens the CrssRS reaction and produces little or no HtrA and HtrB quality control proteases. As a result, degradation of the α-amylase secreted by the Sec-dependent secretion pathway is decreased, increasing the extracellular α-amylase activity. However, when intracellular protein precursor secretion is slower, a greater number of α-amylase protein precursors fail to cross the plasma membrane, which leads to the formation of inclusion bodies that can be detected using SDS-PAGE (Fig. 3b).

To test the above hypothesis, we constructed two recombinant *B. subtilis* strains that overexpress PrsA: WS11QSP, which expresses AmyS with the SP_AmyQ’_ signal sequence, and WS11YSP, which expresses AmyS with the SP_YojL_ signal sequence. After 48 h of fermentation in shake flasks, the α-amylase activities of the culture supernatants of WS11QSP and WS11YSP were 598.8 and 712.1 U mL^−1^, respectively. Therefore, WS11QSP represents a 2.85-fold improvement in extracellular α-amylase activity over WS11QS (210.4 U mL^−1^), while WS11YSP produced only 97% of the α-amylase activity produced by WS11YS (732.0 U mL^−1^). These data indicate a PrsA deficit in WS11QS that is absent in WS11YS, consistent with the suggestion that lowering the secretion rate prevents the overloading of PrsA.

### Reduce inclusion bodies by overexpressing intracellular molecular chaperones

Most of the extracellularly secreted protein is properly folded within the cell so that it can be effectively transported across the cell membrane. Intracellular molecular chaperones play a major role in maintaining this conformational state [15]. GroEL–GroES and DnaK–DnaJ–GrpE are among the most important chaperone complexes, and they generally exhibit different effects on increasing protein solubility [36]. We cannot, at present, confidently predict which molecular chaperone contributes to the successful secretion of a specific protein. Overexpression of a single molecular chaperone can sometimes cause a defect in cell septation and the formation of cell filaments, which are harmful to the bacteria [37], so it is usually necessary to overexpress both GroEL–GroES and DnaK–DnaJ–GrpE.

**Fig. 4:**
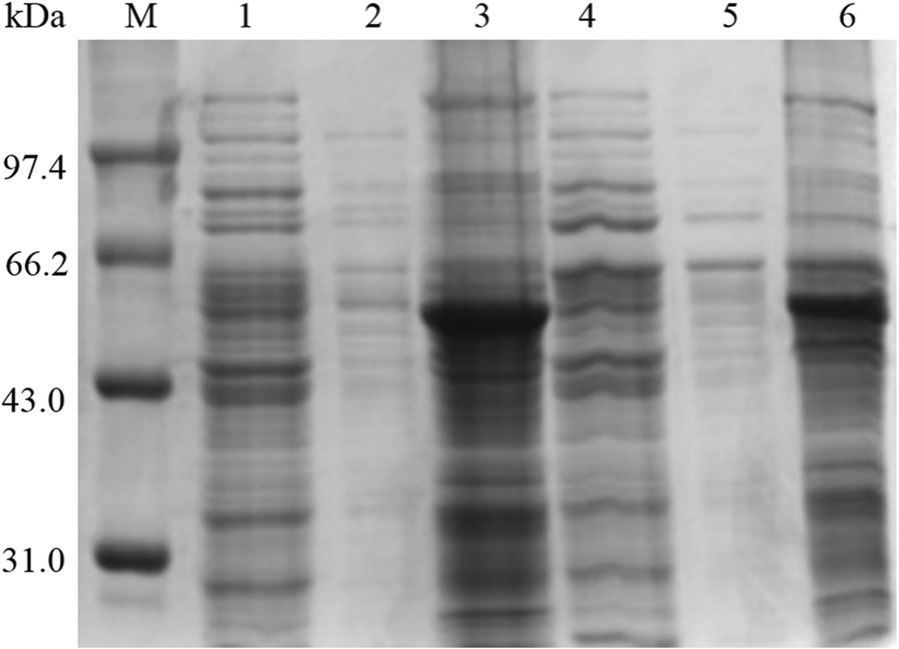
Production of α-amylase by *B. subtilis* strains WS11YS and WHS11YS. SDS-PAGE analysis of the culture supernatants (lanes 1 and 4), cell disruption supernatants (lanes 2 and 5) and cell disruption precipitates (lanes 3 and 6) obtained through shake-flask culture of WS11YS and WHS11YS. Lane M contains the medium molecular weight protein standard

Mogk et al. [38] reported that these two operons are co-regulated by the repressor HrcA, while HrcA activity is regulated by GroE. Inactivation of the *hrcA* gene enables overexpression of both series of intracellular chaperone proteins [39]. Because our laboratory previously constructed WHS11, a WS11 variant in which the *hrcA* gene has been inactivated, this strain was transformed with the expression vector pHYYamyS. After shake-flask fermentation for 48 h, the α-amylase activity of the culture supernatant from WHS11YS was 1039.4 U mL^−1^, which was 1.42-fold greater than that of WS11YS (732.0 U mL^−1^). As seen in Fig. 4, the inclusion body content of WHS11YS was significantly smaller than that of WS11YS, but there were still some inclusion bodies. Wu et al. [40] reported similar results when expressing an anti-digoxigenin single-chain antibody. In their study, inactivating the *hrcA* gene resulted in the coordinated overproduction of GroEL–GroES and DnaK–DnaJ–GrpE, which improved secretion of the antibody, which easily formed inclusion bodies, by approximately 60%.

### High-throughput screening of α-amylase mutants

The heterologous expression level of a foreign protein is related to its primary sequence [41]. Error-prone PCR combined with high-throughput screening technology is an important means to create and screen libraries of enzyme mutants to identify variants with improved properties. In this study, we used error-prone PCR to construct a library of α-amylase mutants through random mutation of the *amyS* sequence. These mutants were screened by determining the diameters of the clear circles formed on 2% soluble starch plates. Because these clear circles were formed via α-amylase activity, the screening assay was able to rapidly identify mutants with enhanced α-amylase activity.

For the sake of convenience, plasmid vector pET24a(+) and host *E. coli* BL21(DE3) were used to express the library of α-amylase mutants. After screening 5000 strains, a single strain that formed the largest transparent circle on the starch plate was selected for verification via shake-flask culture. The protein produced by this strain, which was found to contain the double mutation K82E/S405R (AmySA), had α-amylase activity that was 2.1-fold greater than that of wild-type AmyS.

**Fig. 5:**
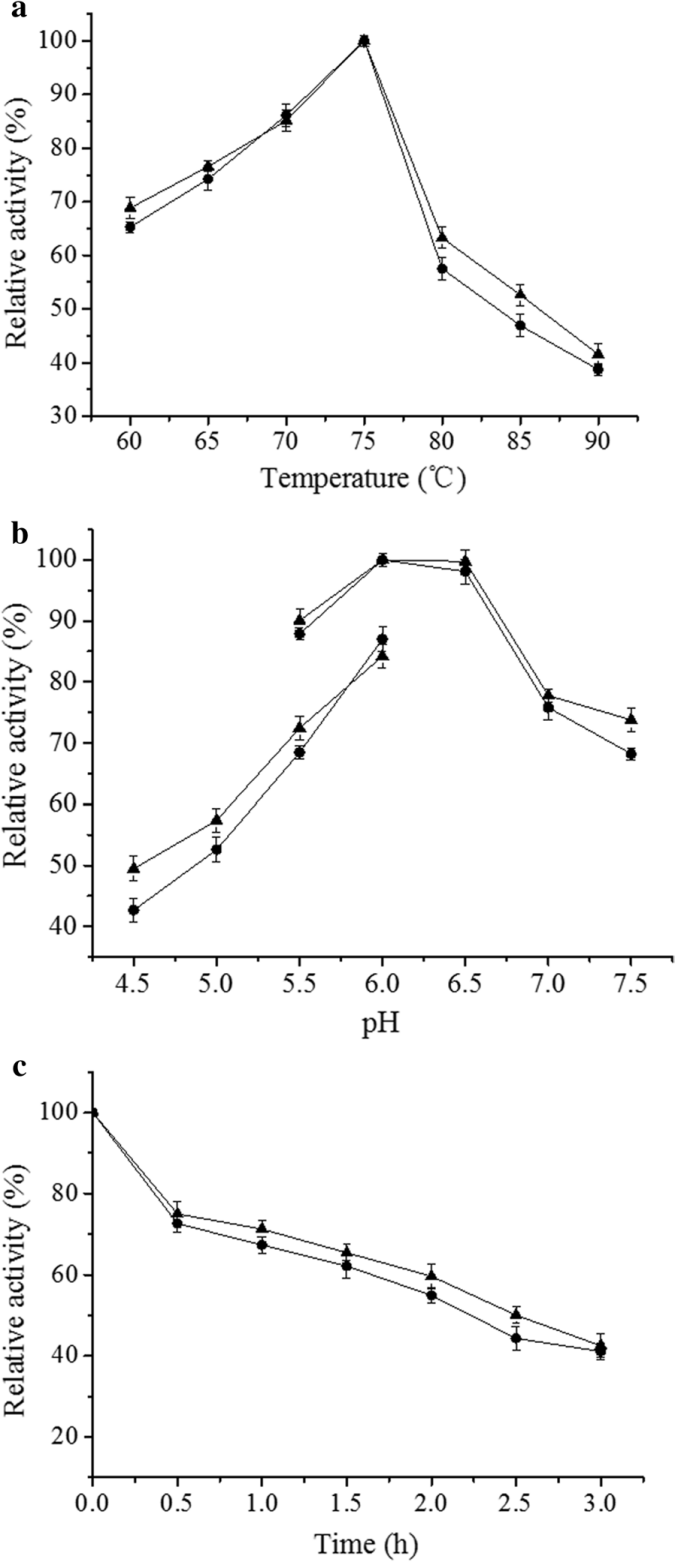
Effect of temperature and pH on the α-amylase activities of AmyS and AmySA. **a** Effect of temperature. **b** Effect of pH. **c** Thermal stability of AmyS and AmySA at 90 °C, pH 6.0, and 1 mM Ca^2+^. Filled triangle, AmyS; filled circle, AmySA. Error bars indicate the standard deviation from the mean of the three experimental data replicates

To compare the enzymatic properties of AmyS and its mutant AmySA, these proteins were produced in *E. coli*, purified, and then characterized. The specific activities of AmyS and AmySA were 28,260 U mg^−1^ and 29,070 U mg^−1^, respectively. As shown in Fig. 5a, AmySA has the same optimum temperature (75 °C) as AmyS. At temperatures > 75 °C, the rate of decline in enzyme activity of AmySA was slightly higher than that of AmyS, but the difference was barely noticeable. The enzyme activities of AmyS and AmySA at 90 °C were 41.6% and 38.7% of that at 75 °C, respectively. As shown in Fig. 5b, AmyS and AmySA shared the same optimum pH (pH 6.0), and at same pH, their activities were greater in phosphate buffer than in citrate buffer. At 90 °C and pH 6.0, in the presence of 1 mM Ca^2+^, AmyS and AmySA showed similar thermal stability. After 3 h, the enzyme activities of AmyS and AmySA remained 42.5% and 41.1%, respectively, of their original activities at 70 °C (Fig. 5c).

To assess the extracellular production level of AmySA in *B. subtilis*, strain WHS11YSA was constructed. This strain, which is otherwise identical to the wild-type α-amylase expression strain WHS11YS, was cultured in a triangular shake flask for 48 h. The α-amylase activity of the supernatant (1496.8 U mL^−1^) was 1.44-fold greater than that of WHS11YS (1039.4 U mL^−1^). It is interesting that the degree of increase obtained using *B. subtilis* as the expression host (1.44-fold improvement) was lower than that observed when using *E. coli* BL21 as the expression host (2.1-fold improvement). This may be due to differences in the physiological characteristics of the two host strains and differences in the expression vectors used in these hosts.

### Scale-up of α-amylase production in a 3-L fermenter

**Fig. 6:**
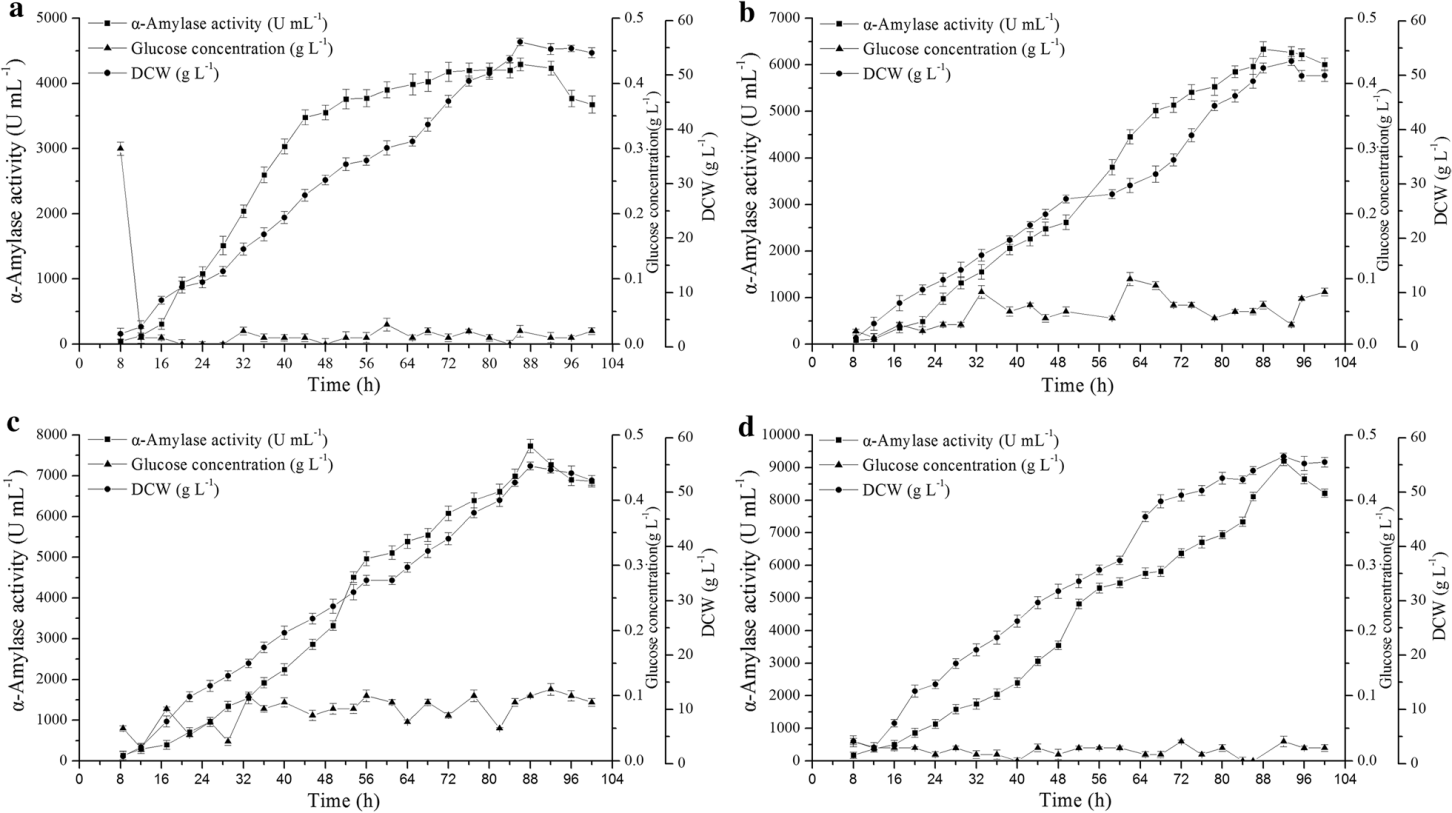
Alpha-amylase production by various recombinant strains in a 3-L fermenter. **a** WS11QS, **b** WS11YS, **c** WHS11YS, **d** WHS11YSA. Error bars indicate the standard deviation from the mean of the three experimental data replicates

In the experiment described above, after 48 h of shake flask fermentation, the α-amylase activities in the fermentation supernatants of WS11YS, WHS11YS, and WHS11YSA were 3.5-, 4.9- and 7.1-fold greater than that of the original strain WS11QS, respectively. To verify whether all manipulations performed to increase activity of α-amylase detected in culture supernatant during the shake flask fermentation were indeed significantly different from that observed for parental strain WS11QS, the strains WS11QS, WS11YS, WHS11YS, and WHS11YSA were subjected to 3-L fermenter fermentation verification (Fig. 6).

When the carbon source in the initial culture was consumed, a feed medium was added. During fermentation in the 3-L fermenter, the α-amylase activities in the fermentation supernatants of strains WS11QS, WS11YS, WHS11YS and WHS11YSA reached peak levels at 86, 88, 88 and 92 h, respectively, and the highest amylase enzyme activities were 4293.9, 6337.8, 7728.9 and 9201.1 U mL^−1^, respectively. These were 20.4-, 8.7-, 7.4- and 6.1-fold greater than the amylase activity in the fermentation supernatant at 48 h of the corresponding strain in shake flask fermentation, respectively. The reason that the highest amylase activity obtained by fermentation of different strains in 3-L fermenter had a different increase than the amylase activity obtained by shake flask fermentation supernatant may be because the fermentation medium used by all strains in the 3-L fermenter fermentation process is our optimal fermentation medium optimized for WS11QS, which may not be the optimum fermentation medium for other strains.

The highest amylase activity in the 3-L fermenter fermentation supernatant of the strain WHS11YSA was 2.1-fold greater than that of the strain WS11QS. To compare this level with those previously reported, the α-amylase activity of the supernatant obtained using WHS11YSA in the 3-L fermenter (9021.1 U mL^−1^) was 1.8-fold greater than that obtained in a 7.5-L fermenter using *B. subtilis* 1A751pSP4 (5086 U mL^−1^) [8], which is the highest heterologous extracellular production level that has been reported for α-amylase from *B. stearothermophilus* in *B. subtilis*.

## Conclusions

High-level expression of *B. stearothermophilus* α-amylase in *B. subtilis* was achieved by screening optimal signal peptides, overexpressing chaperones, and performing random mutagenesis of the amylase gene. After 48 h of shake-flask culture, the α-amylase activity of the supernatant of *B. subtilis* WHS11YSA (1496.8 U mL^−1^) was 7.1-fold greater than that of WS11QS (210.4 U mL^−1^). After incubation for 92 h in a 3-L fermenter, the α-amylase activity that was obtained using WHS11YSA was 9201.1 U mL^−1^. This represents the highest amylase activity ever reported for α-amylase from *B. stearothermophilus* in *B. subtilis*. This high-level expression lays a good foundation for enhanced industrial production of α-amylase.

## Additional file

**Additional file 1: Figure S1.** N-terminal amino acid sequence results of inclusion bodies. (A) Standard mixtures of 19 type PTH amino acids. (B)-(F) is the 1–5 amino acids of inclusion bodies N-terminus. **Table S1.** Primers used in this study.

## Abbreviations

AmyS: α-amylase
AmySA: α-amylase mutant K82E/S405R
GRAS: generally recognized as safe
TF: trigger factor
DCW: dry cell weight
DNS: 3,5-dinitrosalicylic acid
SDS-PAGE: sodium dodecyl sulfate-polyacrylamide gel electrophoresis
