# Supplementary material for: Enhanced extracellular expression of Bacillus stearothermophilus α-amylase in Bacillus subtilis through signal peptide optimization, chaperone overexpression and α-amylase mutant selection
Source: Microb Cell Fact. 2019 Apr 11;18:69. doi: 10.1186/s12934-019-1119-8 (PMC6458788; doi:10.1186/s12934-019-1119-8)
Supplement: Supplementary file 1 — Additional file 1: Figure S1. N-terminal amino acid sequence results of inclusion bodies. (A) Standard mixtures of 19 type PTH amino acids. (B)-(F) is the 1–5 amino acids of inclusion bodies N-terminus. Table S1. Primers used in this study. [file 12934_2019_1119_MOESM1_ESM.docx]

Enhanced extracellular expression of *Bacillus stearothermophilus* α-amylase in *Bacillus subtilis* through signal peptide optimization, chaperone overexpression and α-amylase mutant selection

Dongbang Yao^a,b,c^, Lingqia Su^a,b,c^, Na Li^a,b,c^, Jing Wu^a,b,c,*^

^a^State Key Laboratory of Food Science and Technology, Jiangnan University, 1800 Lihu Avenue, Wuxi, 214122, China

^b^School of Biotechnology and Key Laboratory of Industrial Biotechnology Ministry of Education, Jiangnan University, 1800 Lihu Avenue, Wuxi, 214122, China
^c^International Joint Laboratory on Food Safety, Jiangnan University, 1800 Lihu Avenue, Wuxi, 214122, China

*Correspondence: jingwu@jiangnan.edu.cn

State Key Laboratory of Food Science and Technology, Jiangnan University, 1800 Lihu Avenue, Wuxi, Jiangsu 214122, China.

**SUPPORTING INFORMATION**

**Figure S1.** N-terminal amino acid sequencing results of inclusion bodies. (A) Standard mixtures of 19 type PTH amino acids. (B)-(F) is the 1-5 amino acids of inclusion bodies N-terminus.

**Table S1.** Primers used in this study.


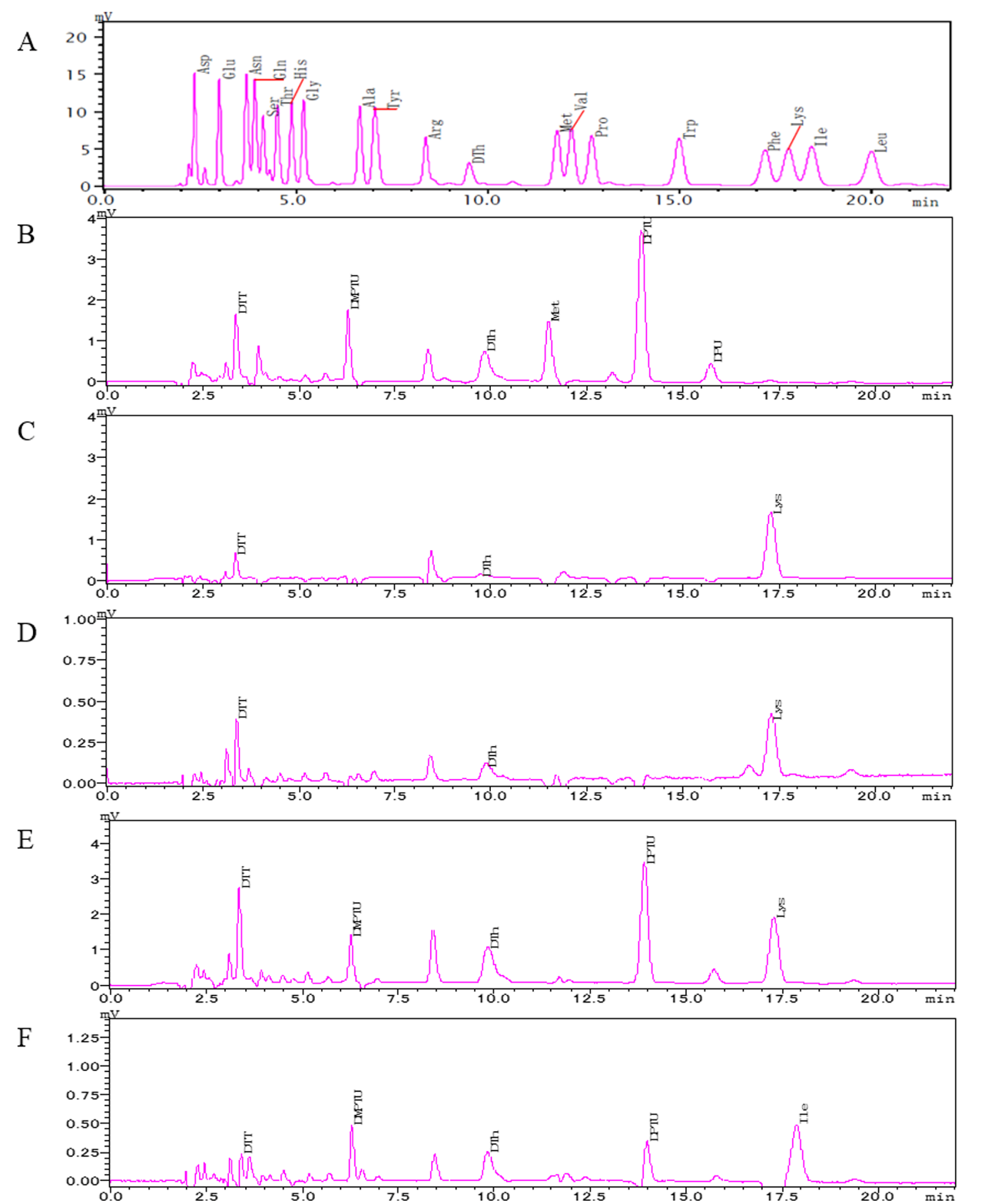


**Figure S1.** N-terminal amino acid sequenced results of inclusion bodies.

(A) Standard mixtures of 19 type PTH amino acids. (B)-(F) is the 1-5 amino acids of inclusion bodies N-terminus.

**Table S1.** Primers used in this study.

| Primers | Sequences (5’-3’) |
| --- | --- |
| pBE1 | ATCGAGCTCATGGCAGCCCCGTTCAAT |
| pBE2 | CCCAAGCTTTTAGCGCGGAACCCACACACT |
| pBE3 | CGCGTCCCTCTCCTT TTGCTTAAGTTCAGAGTAG |
| pBE4 | GGCCGGTGCACATATGGAGCTCGGTACCCTCGAG |
| pHY1 | AAGCTTGGTAATAAAAAAACACCTCC |
| pHY2 | CATGGCTTCAGCACTCGCA |
| pHY3 | CTGCGAGTGCTGAAGCCATGATGGCAGCCCCGTTCAAT |
| pHY4 | GTTTTTTTATTACCAAGCTTTTAGCGCGGAACCCACAC |
| pHY5 | AAGCTTGGTAATAAAAAAACACCTC |
| pHY6 | TCTTGACACTCCTTATTTGATTTTTTG |
| pHY7 | TCAAATAAGGAGTGTCAAGAATGAGAAAAAAGATTACGTTAG |
| pHY8 | TCAAATAAGGAGTGTCAAGAATGAAACTGGCAAAAAGAGTAT |
| pHY9 | TCAAATAAGGAGTGTCAAGAATGAAAAAGAAGATTGTAGCCG |
| pHY10 | GTTTTTTTATTACCAAGCTTTTAGCGCGGAACCCACACACTCA |
| pHY11 | CCCACGTTGTGATTAAAAGCAG |
| pHY12 | AATTCCTGTTATAAAAAAAGGATCAA |
| pHY13 | TTTTAATCACAACGTGGGAGGCCGTCTGTACGTTCCTAAACTAGT |
| pHY14 | CTTTTTTTATAACAGGAATTCCCGGCAGTACCGGCATA |
| pF1 | CATGCCATGGATGGCAGCCCCGTTCAATGG |
| pR1 | CCCAAGCTTAGCGCGGAACCCACACACT |
